# Why do women not use skilled birth attendance service? An explorative qualitative study in north West Ethiopia

**DOI:** 10.1186/s12884-020-03312-0

**Published:** 2020-10-19

**Authors:** Biruhtesfa Bekele Shiferaw, Lebitsi Maud Modiba

**Affiliations:** 1 Department of Health Studies, University of South Africa, Addis Ababa, Ethiopia; 2 Department of Health Studies, University of South Africa, Pretoria, South Africa

**Keywords:** Home delivery, Skilled delivery service, Health system-related factors, Maternal mortality, Client-related factors, Ethiopia

## Abstract

**Background:**

Having a birth attendant with midwifery skills during childbirth is an effective intervention to reduce maternal and early neonatal morbidity and mortality. Nevertheless, many women in Ethiopia still deliver a baby at home. The current study aimed at exploring and describing reasons why women do not use skilled delivery care in North West Ethiopia.

**Methods:**

This descriptive explorative qualitative research was done in two districts of West Gojjam Zone in North West Ethiopia. Fourteen focus group discussions (FGDs) were conducted with pregnant women and mothers who delivered within one year. An inductive thematic analysis approach was employed to analyse the qualitative data. The data analysis adhered to reading, coding, displaying, reducing, and interpreting data analysis steps.

**Results:**

Two major themes client-related factors and health system-related factors emerged. Factors that emerged within the major theme of client-related were socio-cultural factors, fear of health facility childbirth, the nature of labour, lack of antenatal care (ANC) during pregnancy, lack of health facility childbirth experience, low knowledge and poor early care-seeking behaviour. Under the major theme of health system-related factors, the sub-themes that emerged were low quality of service, lack of respectful care, and inaccessibility of health facility.

**Conclusions:**

This study identified a myriad of supply-side and client-related factors as reasons given by pregnant women, for not giving birth in health institution. These factors should be redressed by considering the specific supply-side and community perspectives. The results of this study provide evidence that could help policymakers to develop strategies to address barriers identified, and improve utilisation of skilled delivery service.

## Background

Globally, the maternal mortality ratio (MMR) reduced by 38%, that is from 342/100,000 live births in 2000 to 211/100,000 live births in 2017. However, 295,000 maternal deaths occurred in 2017 worldwide. Unsurprisingly, developing countries accounted for about 86% of the estimated maternal deaths in the same year, with sub-Saharan countries alone accounting for 66% of the maternal deaths [1]. Likewise, though the MMR in Ethiopia reduced substantially from 871 deaths/100,000 live births to 412 deaths/100,000 live births between 2000 and 2016, the MMR is still very high [2]. The overwhelming maternal deaths happened due to haemorrhage, hypertensive disorders, sepsis, abortion, embolism, complications of anaesthesia, and peripartum cardiomyopathy [3, 4].

A variety of clinical and community-level strategies and interventions should be put into practice across the continuum of care to reduce maternal mortality. Among these, skilled maternity care could avert a substantial proportion of women’s death during and following pregnancy and delivery [5–7]. Besides, the availability and provision of emergency obstetric and neonatal care (EmONC) in health institutions, to treat life-threatening complications, is an essential requirement to avert of a significant proportion of the maternal deaths [6, 8, 9]. Based on the United Nations (UN) recommended minimum standard (5 EmONC facilities/500,000 population), the availability of functioning EmONC in heath facility in Ethiopia was found to be only 40%. The met need for EmONC was also very low (18%) [10].

As stated in the Ethiopian mini demographic and health survey 2019, the percentage of live births attended by a skilled provider has substantially increased from 5% in 2005 to 48% in 2019. Nevertheless, the percentage of live births that occurred in health institutions is still low [11]. Several studies conducted in Ethiopia identified multitudinous bottlenecks and challenges to skilled delivery service use though a significant share of the evidences emanates from quantitative studies. Low maternal education, being rural residents, multiparty, lack of awareness of ANC, remoteness /lack of transportation to health institution, inexperience of obstetric complications and care in previous delivery, lack of birth preparedness and complication readiness, poor decision making power women, and low awareness of obstetric care were identified as deterrents to skilled delivery service use [12–17]. Furthermore, a qualitative study conducted in southern Ethiopia revealed that reliance on traditional birth attendant over skilled maternity care provider, low quality of service, disallowing companions to enter labour and delivery room, socio-cultural factors, economic factors, lack of privacy, information gap, poor reception, poor knowledge and skills of staffs, shortage of staff, and negative health facility childbirth experience discerned these factors to be deterrents to skilled delivery service use [18, 19].

Many countries including Ethiopia registered tremendous achievements in majority of the Millennium Development Goals (MDGs), and targets during the 15 year period (2000–2015). Leaders from 189 countries desired to build on the successes of the MDGs and go further, and created an ambitious plan called the Sustainable Development Goals (SDGs). The new sets of goals with specific targets, i.e. SDGs that lasts between 2015 and 2030, has been developed with 17 goals. These goals build on the gains and successes of the MDGs and further accelerate the global development. Hence, the international community is on a crusade to reduce MMR to less than 70/100,000 live births by 2030, which is a target set to be achieved during the SDGs period [20]. Since Ethiopia adopted the SDGs and aspires to achieve these goals and targets the Ethiopian government has set a target of reducing MMR from 420 to 199/100,000 live births between 2015 and 2020, in the national five years health sector transformation plan. Thus, a set of high impact interventions and strategies including family planning, focused ANC, skilled birth service, early postnatal care, improved health facility coverage, and expansion of emergency obstetric services are being implemented to reduce maternal mortality [21].

Many quantitative studies have been conducted in Ethiopia to find out why mothers prefer for home childbirth. A few qualitative research studies were also undertaken to explore the barriers and deterrents for skilled delivery service use in the study districts. Therefore, the objective of this study was to make an in-depth exploration and describe why pregnant mothers choose for home childbirth in North West Ethiopia.

The results of this study aim to provide inputs to the development of strategies to improve skilled delivery service uptake, and corresponding declines in maternal morbidity and mortality.

## Methods

This study was part of a bigger research project by the researchers (Biruhtesfa Bekele and LM Modiba) and details of the research methods have been published elsewhere [22].

### Study setting

The research was done in two rural districts (Womberema and Burie Zuria) of the Amhara region in North West Ethiopia. Primarily, the Amhara regional state was selected for this study because of the low coverage of skilled delivery service (27.1%) [2]. By selecting this region, which was identified as having low performance in skilled delivery service, the study intended to inform designing of strategies that will help in improving utilization of skilled delivery care. By doing so, an in-depth understanding of reasons for poor or no utilization of skilled delivery care, was critical. As data acquired from regional routine health management information system (HMIS) reports evidenced, there was a variation in coverage of skilled delivery performance among health institutions and district health offices in the study region. Though several districts had low performance, a few of them were performing well. Our study focused on the districts with low performance in skilled delivery care. Well-performing districts in skilled delivery care were excluded from the study, because this research was not a comparison study.

**Fig. 1:**
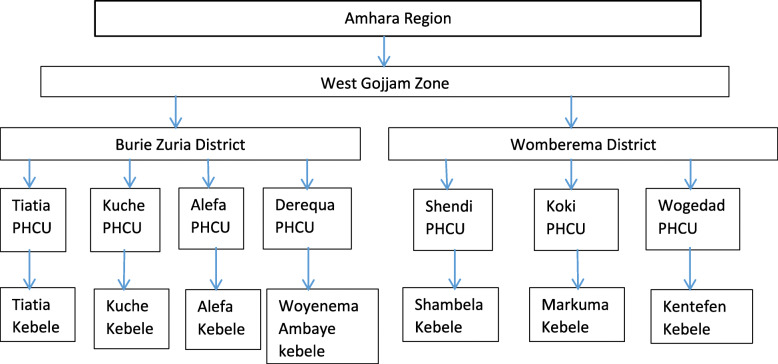
Sampling procedure of the study sites

Each of the study districts comprised of 20 rural kebeles, 4 health centers, and 20 health posts. We purposively selected 7 kebeles for this study, 4 of them were from Burie Zuria District and the remaining three kebeles were from Womberema district. The detail of the sampling of the research sites is portrayed in Fig. 1.

### Study design, participants and sampling procedure

A qualitative descriptive explorative study design was employed to explore and describe why pregnant women in North West Ethiopia do not use skilled delivery service. A descriptive qualitative research was done because the phenomenon of interest in the current study has been well-defined, and because of the need to describe the subject of study accurately and present a detailed picture or accounts of the phenomenon of interest [23]. In view of this, the coverage of utilization of skilled delivery care in the study districts and region has been well known and many quantitative researches have been conducted on the subject under study. Therefore, this descriptive qualitative research was done aimed at describing and presenting a detailed accounts or pictures on why mothers do not use skilled delivery care in the study areas.

To capture reasons for home childbirth, data were collected from pregnant women, and mothers who delivered in the past one year. The research participants were purposively included in the study if they had previously given birth once or more at home, in health institution or both. The study subjects were identified through health extension workers (HEWs), who work in the selected kebeles and serve the community by providing basic promotive, preventive, and selected curative services. We also corroborated whether the selected participants fulfilled the inclusion criteria or not. Before embarking on the actual data collection, we established a good rapport with the study subjects, and this enabled us to win their trust which helped to obtain the information needed. The researchers also asked the participants a series of follow-up and probing questions, after posing the main question. This helped to take the discussions to a deeper level and obtain required information.

### Data collection

Two experienced qualitative data collectors who were graduates of health science and social science conducted the data collection. A focus group discussion was used to capture the data. The researchers prepared and used a semi-structured FGD guide to guide the group discussions. We conducted pretesting of the preliminary FGD guides with each of the research participants who were excluded from the actual data collection. This helped in estimating time required to conduct the interviews and FGDs, to refine the interview guides and questions, to check appropriateness of the data capturing procedures and to familiarise the researcher with the data recording equipment (audiotape recorder). All the FGDs were conducted in Amharic, which is the official business language of Ethiopia and is widely spoken in the region. The data collectors conducted the FGDs in the compounds of the health posts because of health posts’ accessibility to research participants and avoid any disturbance from non-participants because the health posts had a fence. We made sure that the HEWs were not around while the FGDs were underway. The FGDs lasted between 60 to 90 min. The data collectors took field notes during FGDs, expanded notes after each FGD sessions, and shared with researchers and data collectors. All the FGDs were digitally recorded with participants’ consent using two audiotape recorders, one was used as a backup, in case the other audio tape recorder failed. We conducted FGDs separately with pregnant women (7 FGDs) and mothers who delivered within 12 months (7 FGDs). The researcher recognised that no new idea or insight emerged after conducting five FGDs with pregnant women and five with mothers who delivered a child within one year. This revealed data saturation and was confirmed as such, with a final additional of two FGDs with pregnant women and two with mothers who delivered a child within one year. Those two final FGDs were added only for sake of confirmation of point of saturation; otherwise, it had no any relevance.

### Data analysis

An inductive thematic analysis approach was employed to analyse the qualitative data. The translated data were exported onto Atlas ti version 7 software to efficiently store, organise, manage and reconfigure the data to enable human analytic reflection. The current study adhered to data analysis steps which included reading, coding, displaying, reducing, and interpreting.

The data analysis was initiated in the field before completion of data collection. The researcher listened to the audio files and read the expanded field notes and transcripts after each FGD session is completed and the transcribed data were ready to use. The audiotape records of the FGDs were transcribed and the data collectors prepared the interview transcripts for analysis expanded the field notes. The researcher translated the Amharic transcripts directly into English. To ensure accuracy of the translation, a colleague of the researcher, who has high level of proficiency in both English and Amharic language, checked the consistency of the translation. The engagement of the researcher in the translation and partly in the transcription of the interviews helped to familiarise and acquaint himself with concepts. Moreover, the whole process of analysing the data in advance helped to make necessary revisions and refinements before subsequent FGD sessions took place.

The transcribed data (transcripts) were imported to the Atlas ti version 7 data analysis software as a primary document, using the assign command in the main menu of the Atlas ti. The researcher labelled the coded texts with words that explain the text description. The techniques employed for coding were open coding, quick coding and coding by list. The researcher used the open coding command when coding for the first time. Coding by list helped to assign existing codes to a selection and quick coding was employed to assign currently selected codes to consecutive text segments. The codes were then examined to form categories and sub-categories. The study explored patterns of categories to discover emerging themes.

The study credibility, where the results of the research are closely related to reality, was achieved through prolonged engagement, triangulation, peer debriefing, and member check. Prolonged engagement involves establishing adequate contact with the participants and the context with the objective of acquiring data the researchers needs. We spent adequate period of time in the study areas with the research participants in FDGs and this enabled the researchers to acquire adequate understanding of the contexts and to establish rapport with the participants. We also collected separate data from pregnant women and mothers who delivered a child recently, who have different childbirth experiences, to conduct data triangulation. Besides, peer debriefing was held with colleagues from universities who were experienced in qualitative research, and presentation of the results and interpretations of the data were made. This helped to avoid bias and misinterpretation of the data and unfolded aspects of the research that remained covert. Member check was also used to ensure the study credibility in which study participants were allowed to validate whether the researchers’ interpretations were a good representation of the participants’ realities.

### Ethical considerations

To conduct this study, ethical clearance was secured from the UNISA Department of Health Studies Higher Degrees Committee and Amhara Regional Health Bureau Research and Laboratory Department. The research got letters of support from regional health bureaus and zonal health departments to get access to the study sites. Written informed consent was acquired from participants who could read and write and fingerprints were used for participants unable to read and write. Confidentiality was ensured by removing all names and addresses of participants from the data collecting tools. The information that the participants provided was also kept confidential and used only for the research. Only codes were used to identify participants. Data collected were kept in the strictest confidence; they were not made public. The audio files, saved on memory cards, were also erased after the completion of the research. Only aggregated demographic information was reported to maintain anonymity.

## Results

### Basic characteristics of the participants

One hundred thirty-three women (133) were involved in the FGDs. Of these, 62 women took part in the pregnant women group while the remaining 71 took part in the group of mothers who delivered within one-year. Majority of the participants were in the age category of 20–29 years while about 100% were married and Orthodox Christians. Virtually all participants were unemployed and two-third of them never went to school. Furthermore, about half of them got pregnant at least three times. Majority of the participants, pregnant women (62.9%) and mothers who delivered within one year (57.7%), had between 1 and 3 children (Table 1).

**Table 1:** Demographic characteristic of participants

Characteristics		Mothers who delivered within one year (*n* = 71)		Pregnant women (*n* = 62)	
		Frequency	Percentage	Frequency	Percentage
Age	17–19	3	4.2	1	1.6
	20–24	20	28.2	16	25.8
	25–29	27	38.0	22	35.5
	30–34	16	22.5	17	27.4
	> = 35	5	7.0	6	9.7
Religion	Orthodox	71	100	62	100
Marital status	Divorced	3	4.2	0	0.0
	Married	68	95.8	62	100
Occupation	Employed	1	1.4	1	1.6
	Unemployed	70	98.6	61	98.4
Education	No education	46	64.8	40	64.5
	Primary	10	14.1	7	11.3
	Secondary	7	9.8	7	11.3
	High school and above	8	11.3	8	12.9
Gravidity	1–3	35	49.3	31	50.0
	4–6	31	43.7	28	45.2
	> = 7	5	7.0	3	4.8
Parity	0	0	0.0	8	12.9
	1–3	41	57.7	39	62.9
	4–6	27	38.0	14	22.6
	> = 7	3	4.2	1	1.6
Delivery place of last child	Home	27	38.0	41	66.1
	Health Facility	41	57.7	12	19.4
	Health post	3	4.2	1	1.6

The current study identified a myriad of health system related and client-related factors that deterred women from using skilled delivery service, which can be classified into two major themes. The themes were health system-related factors and client-related factors.

The health system-related factors included the following sub-themes: low quality of service, lack of respectful care, and inaccessibility of health facility.

Within the major theme of client-related, the sub-themes that emerged were socio-cultural factors, fear of health facility childbirth, the nature of labour, lack of ANC during pregnancy, lack of experience in giving birth in health institutions, low awareness of early care-seeking behaviour. Details of the sub-themes that relate to the two major themes are as seen below.

### Health system-related factors

#### Low quality of service

The experience of receiving poor skilled delivery service from health facilities during previous deliveries was a deterrent to use skilled delivery service. Those women who experienced such poor services assumed the same thing could occur to women again in their subsequent pregnancies. Hence, they were not interested in using skilled delivery service.

*“In my previous pregnancy, my labour started at dusk...I spent the whole night labouring and the next day, Sunday morning, my father took me to the health centre. He told the health worker that I was in labour and asked the health worker to provide maternity care for me. But, the health worker said that he was off duty that day. My father became angry and argued with him...Ever since, I have not been interested to visit the health centre because I believe that they can do nothing for me.”* A lactating mother from Kentefen kebele

*“What deterred women from giving birth in health institutions previously was the reason that even if we went to the health centre seeking skilled delivery care, nobody would be there to treat and care for us.”* A pregnant woman from Woynema Ambaye kebele

The bad reputation of health facilities that prevailed in the communities, deterred women from utilising skilled delivery service. The research participants believed that the skilled delivery service provided at the health institutions was not that different from services that used to be provided in earlier times. Therefore, women preferred home childbirth. The focus group participants mentioned the following verbatim quotes:

*“...I heard that giving birth in this health centre is a very bad thing.”* A lactating mother from Zalema kebele

*“Women these days also think that the health centre’s service provision is the same as it used to be. Hence, they don’t go to health institution for skilled delivery care because women don’t have adequate information about the service provision.”* A pregnant woman from Tiatia kebele

Poor information provision from maternity care providers was also another reason encouraging home delivery. The FGD participants perceived that the maternity care providers misinformed them about their expected date of delivery (EDD) and failed to explain the meaning and implication of EDD to pregnant women. Thus, women claimed that while they (women) were waiting as per their EDD they would unexpectedly deliver at home before their EDD. This shows that health workers need to clearly explain the exact meaning of EDD to women during ANC visits.

*“The reason I delivered my baby at home was that the maternity care providers misinformed me about my expected date of delivery. I have been regularly visiting the nearest public health center for an antenatal check-up. I just came to the health centre at nine months of gestation. The maternity care providers told me that the gestational age of my pregnancy was nine months. At the same time, they informed me that I might give birth between 37-42 weeks of gestation. However, I gave birth at night on the same day I visited the health centre. That is to say that the maternity care providers already told me that my expected date of delivery would be after some time; however, it did not happen that way.”* A lactating mother from Kuche Town

#### Lack of respectful care

Inhibition regarding parturient mobility and making loud noises during labour and delivery was found to be another reason for not utilising skilled delivery service. The FGD participants mentioned that women were inhibited from moving in ways they wanted to during labour and delivery. Besides, women were not allowed to make loud noises during labour because they would be reprimanded by the maternity care providers.

*“Women think that if they go to the health centre, they might not be allowed to move in ways that would make them comfortable [during labour]. During childbirth, they cannot scream whenever they feel like it. Therefore, they choose to deliver in their home.”* A lactating mother from Kuche Town

*“Women complained that the maternity care providers inhibit labouring mothers from moving around until their labour progresses to the final stage.”* A pregnant woman from Kentefen kebele

The participants claimed that companions were also not allowed to enter the labour and delivery room. The parturient women felt abandoned and alone in the room and the maternity care providers even did not stay with the labouring mothers thus leaving them with no one to support and encourage them. This distressed the labouring women.

*“One person should be allowed in the labour and delivery room because the labouring woman should not suffer alone. Otherwise, the maternity care providers should stay and attend our delivery instead of leaving. Since companions are not permitted to enter the labour and delivery room, it is better to give birth at home than at the health centre.”* A pregnant woman from Kentefen kebele

*“Since companions are not allowed to enter in the labour and delivery room, labouring women get distressed.”* A pregnant woman from Shambala kebele

Our study showed that women were not interested in utilising skilled delivery service because maternity care providers in the health centres were less attentive towards labouring women. These women complained of not getting adequate support from maternity care providers during their labour and delivery. Consequently, the community had negative attitude towards health workers and this prevailing perception about health workers hindered women from utilising skilled delivery service.

*“The health centre staffs loiter around the facility and do not support us. Hence, we visit health facilities only for ANC check-up but we choose to deliver at home.”* A pregnant woman from Tiatia kebele

*“The reason for not giving birth in health institutions..., when we previously visited health facilities for childbirth, the maternity care providers were not concerned about anybody.”* A lactating mother from Markuma kebele

#### Inaccessibility of health facility

Distance to health institution and inadequate transportation facilities were additional main barriers for not utilising skilled delivery service. This was mainly related to poor roads, far away located health facilities, shortage of ambulance for transportation, or late arrival of ambulance to labouring mothers’ village.

*“I delivered my baby at home. I was having labour pain, we dialled to HEW and she came home. The HEW then dialled the health centre asking them to send an ambulance as soon as possible. But during that time, the ambulance was in another kebele providing service and when it arrived at our village, I had already delivered my baby at home. The HEW attended my delivery.”* A lactating mother from Markuma kebele

*“In our village, most women visit the health centre to give birth there except those who live in distant villages.”* A pregnant woman from Tiatia kebele

### Client related factors

#### Socio-cultural factors

Several socio-cultural factors stemmed from the current study as key barriers to use skilled delivery service. The factors are further categorised into sub themes of traditional factors and influence of families and relatives.

##### Traditional factors

Traditional practices were among the socio-cultural factors causing women not to utilise skilled delivery service. Women claimed that home delivery is a traditional and customary practice. Therefore, they tended to adhere to practising these traditions and customs.

Women claimed cultural ceremonies such as brewing coffee and cooking “Injera” perpetually practised in the house of a parturient woman until she gave birth. The cultural beliefs and ceremonies that were practised in the households of labouring women were causes for delays in women seeking skilled delivery service which often ended up with home delivery.

*“They just keep on practising the traditions, customs, and cultural ceremonies. Previously, there was no health facility so women used to deliver at home. Coffee would be prepared and porridge cooked...They do not get porridge at health facility, so why should they go there?”* A lactating mother from Tiatia kebele

*“This is a traditional and customary practice: the tradition of baking injera and making coffee until we give birth in our home.”* A pregnant woman from Woyneye Ambaye kebele

##### Influence of family members and relatives

The presence of families and relatives of parturient women during home labour and delivery and the physical and emotional support they receive from their families was found to be a reason for not utilising skilled delivery service.

*“It is customary to deliver at home. When we go into labour; we call for our families and relatives and are surrounded by them. We deliver at home and do not need to visit a health facility.”* A pregnant woman from Zalema kebele

Elder families, relatives, and significant others, influenced women not to use skilled delivery service and instead encouraged them to deliver at home. Significant others including parents, mothers-in-law, and fathers-in-law discouraged the women from using skilled delivery service and favoured home delivery. The elders believe that ‘Saint Mary’ protects labouring mothers and through her help the mothers deliver their child safely. In case where parturient women were interested in utilising skilled delivery service, elder family members caused delays in women seeking skilled delivery care.

*“The elders (parents, mothers and fathers-in-law...) encouraged women to deliver at home. They also claim that only Saint Mary can help labouring mothers give birth safely without encountering any problem. Thus, they do not advise women to give birth in health facilities.”* A pregnant woman from Shambela kebele

*“Particularly, mothers and fathers-in-laws, husbands, and the community influenced parturient women to spend some time in their home encouraging them to deliver at home. This has caused delays for women interested to visit the health centre, from visiting the facility.”* A lactating mother from Zalema kebele

##### Fear of health facility childbirth

Fear of giving birth in health institution was a main reason for home childbirth. The study participant women said that they feared repeated vaginal examinations and surgical procedures undertaken in health institution. Women would refuse to get undressed and expose their bare bodies for health workers during labour and delivery. Because of this, women refrained from utilising skilled delivery service.

*“We hear that the maternity care providers insert their hands in reproductive organs of women during health facilities visits. So, women fear to visit the health institution for delivery.”* A pregnant woman from Tiatia kebele

*“When we go to the health facility, we think that health workers might suddenly operate on us and they might insert several things in our vagina and uterus.”* A lactating mother from Woynaye Ambaye kebele

*“...the health workers told me that they wanted to check whether my cervix was opened or not. However, I rebuffed their request and preferred to die in my house than allow them to do as they asked. So I went back home.”* A lactating mother from Kuche town

Negative rumours, superstitions and misconceptions about health facility childbirth deterred women from utilising skilled delivery service. Some of the rumours and misconceptions the women indicated were that, giving birth in health institution causes injuries to the birth canal and uterus. The women also had misconceptions about physical examinations made during labour and delivery.

*“Some people told me that the maternity care providers might hurt me (my birth organs) if I gave birth in health institution. I feared that I might end up having trouble in walking normally again or experience difficulty in controlling my urine.”* A lactating mother from Kuche town

Women also feared that maternity care providers who test pregnant women for HIV, during ANC visits labour and delivery might disclose their HIV serostatus to other people. Because of this, women refuse health facility childbirth thereby avoiding HIV test. Moreover, the FGD participants recounted that the community perceived women who often visit health facility, as being HIV positive. Thus, women chose for home childbirth.

*“If I am tested for HIV during labour and delivery, I believe that the maternity care provider who tested me might disclose my results to other people. I know many people who were deterred from giving birth in health institution because of this.”* A pregnant woman from Kuche town

*“When I went to the health institution for my regular ANC follow-up, I would often attend meetings at the health centre. The community used to talk about me, saying I was infected with HIV because they saw me going regularly and attending meetings at the health facility. I heard this from my neighbours who told me to stop visiting the health centre.”* A lactating mother from Shambela kebele

##### The nature of labour

Most participants of the FGD claimed that precipitated labour was their main reason for not utilising skilled delivery service. The women said that their labour was precipitated and lasted only for a short period, from initiation of labour to childbirth. So instead they preferred for home childbirth.

*“I delivered my last baby at home though I had tried to visit the health centre. My labour was fast and precipitated and we did not even have time to call for an ambulance. I delivered my baby at home. ”* A pregnant woman from Markuma kebele

*“It was a fast and precipitous labour. “Saint Mary” came to me quickly [she delivered quickly]; my labour did not last long.”* A lactating mother from Tiatia kebele

The women also stated that the reason they did not give birth in health centre was that their labour started at night and it was not convenient to arrange any kind of transportation.

*“I had no reservation in giving birth at the health centre but my labour started during the night.”* A lactating mother Tiatia kebele

##### Lack of ANC follow-up during pregnancy

The current research showed that lack of ANC follow-up during pregnancy was also identified as additional reason why mothers do not use skilled delivery service. Women who had no ANC follow-up during their pregnancy did not know their gestational age and expected date of delivery. These women did not receive any kind of health education on the benefit of skilled delivery service.

*“The reason mothers don’t use health facility childbirth service is that they did not have ANC follow-up. Therefore, they do not know their gestational age and expected date of delivery; they deliver at home. If they had received ANC follow-up visits, they would have delivered in the health centre.”* A lactating mother from Kuche town

*“Probably, women don’t visit the health institution for skilled delivery care because they do not attend health education sessions.”* A lactating mother from Markuma kebele

##### Lack of experience of heath facility childbirth

The results of this study showed that previous places where women had childbirth influenced their decision of utilizing skilled delivery service during their subsequent pregnancies. Furthermore, women who had no prior experience in giving birth at health facility but had delivered at home, didn’t have the need to use skilled delivery service in their subsequent pregnancies.

*“I think they had no previous experience of using health facility childbirth service, their previous experience is giving birth at home.”* A lactating mother from Shambla kebele

*“Since women don’t have experience of giving birth in health institution, they are not aware of pregnancy and delivery related problems. They get distressed thinking about health facility childbirth.”* A lactating mother from Tiatia kebele

##### Low knowledge and poor early care seeking behaviour

Lack of awareness about the benefits of skilled delivery service was identified as another reason for not utilising skilled delivery service. The FGD participants stated that women delivered their baby at home because they did not receive health education on the benefits of utilising skilled delivery service.

*“Women delivered their baby at home due to low knowledge on the benefits of skilled delivery service. They did not receive health education thus they are not aware of the benefits of skilled delivery service even though it is good to give birth in health facility.”* A lactating mother Kentfen kebele

*“Since women don’t have knowledge on the benefit of skilled delivery care and the community did not receive health education, women delivered their baby at home. I delivered at home previously because none of us knew about the benefit of skilled delivery service.”* A pregnant woman from Markuma kebele

Poor early care-seeking behaviour of the community was also identified as a reason for home childbirth. This was manifested by the failure of women to seek skilled delivery service unless they encountered health problems and/or obstetric complications. The women recounted that they would have sought skilled delivery service if they had faced health problems and/or obstetric complications during their labour and delivery. Furthermore, the community and families of the parturient women failed to take them to health institution as soon as their labour started. Consequently, the women ended up delivering their baby at home and their families expected that they could have a safe childbirth in their house. However, some families were compelled to take the labouring women to health institution only when they got seriously sick or their labour got complicated.

*“We could have sought for skilled delivery service from health facility if our labour had been prolonged and if we did not give birth after a repetitive coffee ceremony in our house.”* A lactating mother from Woyneye Ambaye kebele

*“Women visited health facilities only when they encountered complications and had health problems. Otherwise, they prefer for home childbirth.”* A lactating mother from Kentefen kebele

*“The reason we do not give birth in health institution is that our families do not take us immediately as our labour starts, but only when we get seriously ill/ or get some complication. We do not visit the health facility as soon as our labour starts.”* A pregnant mother from Shambela kebele

## Discussion

This study set out to make an in-depth exploration of why mothers in North West Ethiopia do not use health facility services for childbirth. The study identified several client and health system-related factors that account for pregnant women not using health facility service for childbirth.

This study indicates the experience of women who received poor health facility services during previous deliveries they had. Consequently, these women believed that the same thing could occur to women in subsequent deliveries if made at health facility and a reason that deterred them from using health facility childbirth services. The quality of services provided for women in their last childbirth determined the childbirth place in their subsequent deliveries [24]. The women’s or their families’ perception of poor services at the health facility also discouraged them from giving birth in health institution [25].

Disrespectful treatment of women was also found to be a critical reason that encouraged home delivery. The inhibition of parturient mobility during labour deterred women from utilising skilled delivery service. This may have adverse birth outcomes and decrease women’s satisfaction of facility childbirth services [26]. Besides, women received reprimands from maternity care providers for making loud noises during labour and delivery. This finding resonates with studies conducted in many countries [27, 28]. However, freedom of movement during labour facilitates progress of labour and enhances the satisfaction of women in facility childbirth [26, 29].

The current study also reveals that disallowing companions into the labour and delivery rooms and leaving women on their own, deterred the women from utilising skilled delivery service and Roro, Hassen, Lemma, Gebreyesus, and Afework corroborate this finding. Support, care and companionship were highly cherished and valued by the participants [30]. However, these didn’t exist at the health facilities. The provision of support by companions of the women’s choice during labour and delivery had positive effects on the women’s satisfaction with the overall birth experience [31].

The negative perception of communities about health facility service provision prevented women from utilising skilled delivery service. Women thought that the provision of skilled delivery service in the health facility was the same as what it used to be previously. Negative childbirth experiences of women and negative perceptions of health worker treatment, low expectation of health service provision by facilities, and poor reputations of these facilities in the communities have dwindled women’s trust in the health system and sustained high rates of home delivery resulting in negatively influencing decisions to utilize delivery services of health institution in the future [28, 32].

Lack of supportive care from maternity care providers was one of the reasons from among many for home childbirth. The participant women accounted that they had no interest in utilising skilled delivery service because health workers were usually unconcerned about the clients and they already had a negative reputation within the communities. This finding was consistent with a study conducted in Rural Zambia [33]. In a study conducted in Tanzania, women were chagrined with the attitude of midwives due to their neglect and lack of support to pregnant women. The husbands who had accompanied their wives to the health facility resonated with the women’s feelings and blamed the maternity care providers for negative birth outcomes. Other community members also shared similar concerns [32].

Women perceived misinformation of their EDD by and failure of health workers to enlighten them on the meaning of EDD is a cause for not utilising skilled delivery service. This finding is in line with a study conducted in South Africa [34]. Health care providers’ failure to inform women about the meaning of EDD emerged as a barrier to access skilled delivery care. Women delivered in their home or on the way to the health facility even though they had intentions of delivering at health facility but lacked knowledge on the concept of EDD. Thus, they could not prepare themselves for health facility childbirth as they were unable to predict their EDD [35, 36].

Distances to health institution, unavailability of ambulance services, and late arrival of ambulances were also additional barriers to access skilled delivery service. Several studies corroborated this finding [36–39].

Several socio-cultural factors were identified by the current study as reasons for home childbirth. Some of these were perpetuation of old traditions and customs. These factors influenced women’s health care decision-making [40]. The perpetuation of old traditions and customs were among the socio-cultural factors for not utilising skilled delivery service. The finding of this study are in congruence with studies conducted in Ethiopia and Bangladesh [18, 37, 41].

Cultural beliefs and ceremonies such as continuous brewing of coffee and frequent baking of “injera” in the home of a parturient woman caused delay in seeking skilled delivery care and often ended up with the woman delivering in her home. The woman turned to health institution, which in most cases is late, if she had difficulty during her labour [30, 42]. The presence of families and relatives in the parturient women during labour was also another reason for home childbirth. They gathered to provide physical and emotional support and encourage and pray for the parturient woman wishing her safe childbirth in her home [18, 25, 42]. Furthermore, elder family members and relatives influenced the woman not to use health facility childbirth service and instead encouraged her for home childbirth. This finding was consistent with studies conducted in Africa and Asia [25, 33, 37]. Fear of giving birth in health institution was among the client related factors and thus reason for not utilising skilled delivery service. The feeling of dread steamed from health facility practices of repeated vaginal examination, women forced to expose their body, surgical operation procedures, being left alone in the labour and delivery room and the fear dying at the health facility. Several studies corroborated this finding [25, 41, 43–45].

Moreover, the current research showed that women’s fear of the disclosure of their HIV status by health workers was a barrier to having health facility childbirth which could result in social, psychological, physical, and economic consequences [45]. The community also perceived women who often visited health facility, as being HIV positive. A study conducted in rural Kenya reaffirmed this misconception in its finding that the community had a perception of health facility childbirth was apt to women with health problems such as HIV. This also contributed to low skilled childbirth coverage [46].

The overwhelming number of study participants claimed that precipitated labour was a main reason for home childbirth. This finding was in congruence with studies conducted in Malawi, Nigeria, and Nepal [47–49].

Rumours and misconceptions about health facility childbirth services was a deterring factor for women from utilising skilled delivery service. Some of the rumours and misconceptions claimed that women who had health facility childbirth had injuries of the birth canal and uterus. This finding was supported by studies conducted in Southern Ethiopia and rural Bangladesh [18, 41].

Women’s labour usually happened at night and it was inconvenient to arrange transportation services. Thus, this was another reason that encouraged home delivery. Researchers corroborated this finding in their studies, stating that labour started at night and families either did not want to travel to health institution and instead the women delivered at home or started the journey to the health facility but the woman gave birth in transit [25, 47].

Lack of ANC service during pregnancy emerged as a reason for home childbirth. The participants explicated that women who had no ANC follow up did not know their gestational age, their EDD, and did not receive information on the benefit of health facility childbirth. Hence, they chose for home childbirth. Several studies conducted in various countries confirmed this finding [15, 44, 45, 50, 51].

The current study pointed out that previous place of childbirth of earlier pregnancies influenced utilisation of skilled delivery care for subsequent pregnancies. Women, who had no prior experience in using health facility childbirth service because they had been giving birth at home, did not show interest in using health facility childbirth service. This shows that earlier places of delivery influence decisions in choosing childbirth place for following births. Especially previous positive experience with home delivery was a common reason for home childbirth. If the previous place was good, it was more likely that the same childbirth place will be considered for their next births [25, 52, 53].

Poor awareness of women of the benefit of skilled delivery service was also identified as a reason for home childbirth. Knowledge of women regarding the benefit of skilled delivery service could influence women’s perceived need for health facility childbirth. This finding was in line with the finding of studies conducted in Ethiopia, Bangladesh and Nepal [19, 41, 54].

Women believed that they would seek skilled delivery service only when they encountered health problems and complications; otherwise, they would prefer for home childbirth. Studies carried out in Nepal and Bolivia corroborated that women and their families did not think it was necessary to visit health institution for normal delivery until and otherwise the women experienced serious health problems [36, 55].

Numerous quantitative studies have been conducted in Ethiopia on the subject of interest but only a handful of exclusive qualitative studies have been conducted for in-depth exploration of reasons in not using skilled delivery health care. This large qualitative study has contributed substantially towards gaining insights and in-depth understandings of the reasons for home childbirth and added new evidence to the body of knowledge. However, the study has some limitations. Firstly, the findings might not be generalizable because the research was done in only two districts of the 15 districts in West Gojjam zone but they might be transferable to other settings of similar characteristics. Secondly, inclusion of well-performing districts (in skilled delivery care) in the study, would have allowed comparisons of findings in two contexts. Besides, this study cannot be an exception from receiving critiques that usually arise in qualitative research often on sample size, interpretation, and bias. Nevertheless, the rich description of sample and point of saturation, data collection methods, the process of analysis, and methods used to establish the trustworthiness of the research, demonstrate transparent nature of the research and debunked this criticism.

## Conclusion

Under client and health system-related themes several factors emerged as reasons for not utilising health facility childbirth service.

Our findings suggest that:

i) designing social behavioural change communication interventions, providing health education, and undertaking advocacy and social mobilisation activities is the mainstay to improve communities’ perceptions and awareness regarding maternal health;
ii) devising interventions that engage elder families and relatives and significant others, to improve their level of awareness regarding skilled delivery care would be critical to redressing barriers related to these group of the community;
iii) developing and implementing continuous quality improvement interventions, based on the provision of care and experience of care model, is very crucial to improve the quality of skilled delivery care and ensure effectiveness of care [56];
iv) improving the interpersonal skills of maternity care providers;
v) humanising delivery care so as to allow companions into the labour and delivery room and creating a home-like environment for women, in health institution;
vi) developing a mechanism to improve and promote positive reputation of health institution;
vii) marketing health service vis-à-vis maternal health services and social support provided in health institution so as to purge some of the barriers related to women’s fear and misconception of delivery services provided in health institution. This includes adequately informing women about maternity care procedures and succinctly explaining when to have surgical procedures (if necessary) and encouraging the pregnant woman to visit the maternity ward before her labour starts;
viii) easily accessing health facility and availing ambulance to transport pregnant women are important. This comprises efforts of making maternity waiting homes available and developing communication mechanisms to ensure timely arrival of ambulances.
  The results of this study provide contextual evidence that could help policymakers develop strategies to address barriers identified and improve utilisation of skilled delivery service.

## Data Availability

The data analysed are available from the corresponding author on a reasonable request.

## Abbreviations

ANC: Antenatal care
EDHS: Ethiopian Demography and health survey
EDD: Expected date of delivery
FGD: Focus group discussion
MMR: Maternal mortality ratio
PHCU: Primary health care unit
HEW: Health extension worker
UNISA: University of South Africa
